# Supplementary material for: A Transcriptomic Analysis of Head and Neck Squamous Cell Carcinomas for Prognostic Indications
Source: J Pers Med. 2021 Aug 11;11(8):782. doi: 10.3390/jpm11080782 (PMC8399099; doi:10.3390/jpm11080782)
Supplement: Supplementary file 1 [file jpm-11-00782-s001.zip › jpm-1309345-supplementary.pdf]

Supplementary Materials: Transcriptomic Analysis for Prognostic Value in Head and Neck Squamous Cell Carcinoma

Supplementary Table S1:
Overexpression of 10 genes with Cox's hazard ratio > 1.5.

Supplementary Table S2:
Overexpression of 10 genes with Cox's hazard ratio < 0.6.

Supplementary Table S3:
Three consensus biomarker candidates.

Table 1: The top 10 genes overexpressed with poor prognosis in the TCGA's HNSCC (ranked by adjusted *P* value)

Gene ID	Gene Description	Kaplan-Meier survival		Univariate		Multivariate	
		FDR *P* value	Bonferroni *P* value	HR*	95% CI	HR*	95% CI
DKK1	dickkopf WNT signaling pathway inhibitor 1	3.8×10^{-6}	0.001	2.266	1.666-3.082	2.135	1.559-2.924
CAMK2N1	calcium/calmodulin-dependent protein kinase II inhibitor 1	1.5×10^{-5}	0.002	2.101	1.572-2.809	2.007	1.490-2.704
STC2	stanniocalcin 2	1.5×10^{-5}	0.004	2.147	1.578-2.921	2.075	1.515-2.843
PGK1	phosphoglycerate kinase 1	2.4×10^{-5}	0.006	2.127	1.563-2.895	2.046	1.498-2.795
SURF4	surfeit 4	6.2×10^{-5}	0.006	2.055	1.531-2.757	2.089	1.543-2.829
USP10	ubiquitin specific peptidase 10	7.9×10^{-5}	0.012	2.083	1.532-2.834	2.119	1.551-2.895
NDFIP1	Nedd4 family interacting protein 1	1.1×10^{-4}	0.017	2.031	1.502-2.746	2.027	1.483-2.771
FOXA2	forkhead box A2	1.6×10^{-4}	0.018	1.976	1.479-2.640	1.914	1.426-2.569
STIP1	stress-induced-phosphoprotein 1	1.8×10^{-4}	0.029	1.958	1.463-2.621	1.957	1.451-2.640
DKC1	dyskeratosis congenita 1, dyskerin	2.8×10^{-4}	0.042	2.046	1.490-2.808	1.837	1.332-2.534
Selection criteria:<br>Kaplan-Meier Bonferroni-adjusted $P < 0.05$<br>Cox's univariate and multivariate $HR > 1.5$<br>(* Cox's model: $P < 0.001$)							

Table 2: The other top 10 genes overexpressed with better prognosis in the TCGA's HNSCC (ranked by adjusted *P* value)

Gene ID	Gene Description	Kaplan-Meier survival		Univariate		Multivariate	
		FDR *P* value	Bonferroni *P* value	HR*	95% CI	HR*	95% CI
ZNF557	zinc finger protein 557	4.7×10^{-6}	0.001	0.465	0.348-0.619	0.499	0.372-0.669
ZNF266	zinc finger protein 266	5.2×10^{-6}	0.001	0.474	0.355-0.632	0.453	0.338-0.607
IL19	interleukin 19	6.5×10^{-6}	0.002	0.472	0.351-0.635	0.459	0.340-0.619
MYO1H	myosin 1H	1.4×10^{-5}	0.003	0.468	0.347-0.632	0.467	0.344-0.634
FCGBP	Fc fragment of IgG binding protein	4.8×10^{-5}	0.008	0.484	0.359-0.653	0.496	0.366-0.674
EVPLL	envoplakin-like protein	7.5×10^{-5}	0.013	0.490	0.363-0.661	0.494	0.364-0.672
PNMA5	paraneoplastic antigen like 5	3.0×10^{-4}	0.017	0.499	0.371-0.671	0.481	0.357-0.650
IQCN	IQ motif containing N	1.5×10^{-4}	0.020	0.500	0.371-0.673	0.483	0.356-0.654
NPB	neuropeptide B	2.6×10^{-4}	0.027	0.460	0.328-0.646	0.457	0.324-0.646
CALML5	calmodulin like 5	2.0×10^{-4}	0.039	0.510	0.379-0.686	0.493	0.364-0.667

Selection criteria:
Kaplan-Meier Bonferroni-adjusted *P* value < 0.05
Cox's univariate and multivariate HR < 0.6
(* Cox's model: $P < 0.001$)

Table 3: The consensus between the TCGA and GSE65858 cohorts in Kaplan-Meier survival and Cox's model

Gene Symbol	KM *P* value		FDR-adjusted *P* value		Cox's univariate HR	
	TCGA	GSE65858	TCGA	GSE65858	TCGA	GSE65858
CAMK2N1	2.97×10^{-7}	6.87×10^{-3}	1.63×10^{-5}	0.038	2.101	1.814
CALML5	5.87×10^{-6}	4.75×10^{-3}	1.97×10^{-4}	0.035	0.510	0.541
FCGBP	1.21×10^{-6}	0.01	4.83×10^{-5}	0.039	0.484	0.573

(FDR: false discovery rate; HR: hazard ratio)

Supplementary Figure S1:
The gene NDFIP1, one of our 20 preliminary candidates, has a *P* value (around 0.05) at 50% quantile cutoff, achieving a *P* value of 2.62×10^{-6} at 70% quantile cutoff.After the FDR correction, NDFIP1 still has *P* value of 1.07×10^{-4}. However, NDFIP1 could not pass the validation by using GSE65858 cohort (n = 270). NDFIP1 has KM *P* value less than 0.05 in GSE117973 cohort (a small HNSCC dataset, n = 87).

Supplementary Figure S2:
A head-to-head comparison of Kaplan–Meier estimates from TCGA HNSCC and GSE65858.

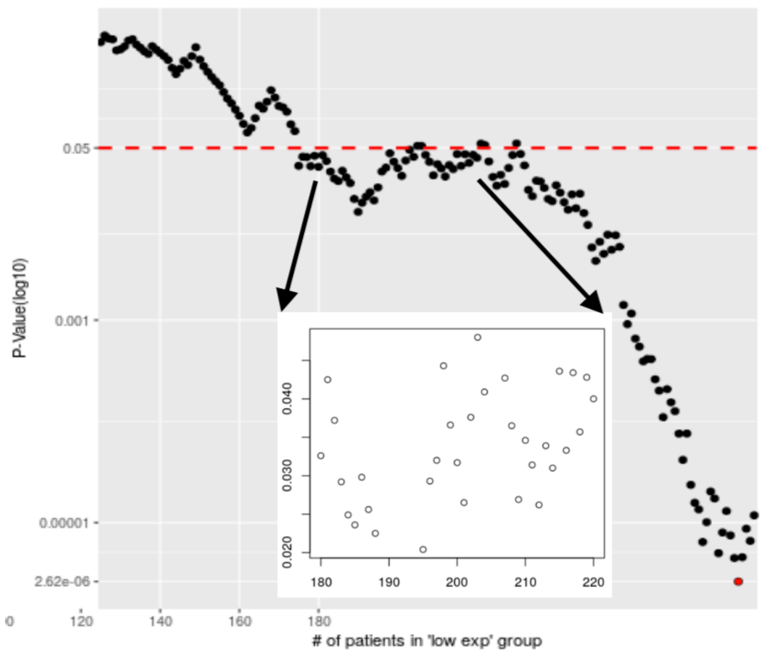

Figure S1. Under cutoff-finding procedure of Kaplan-Meier analysis, the *P*-value plot of gene "NDFIP1" shows: (1) 70% of *P* values is < 0.05; (2) the median-cut zone (zoom-in and revealed in inset box) has a "W"-like distribution; (3) sliding-window cutoff selection could find it's optimized *P* values (far less than 0.001) while a median cut might yield *P* value $>= 0.05$.
(x-axis: grouping by person number; y-axis: *P* value in log10 transformed)

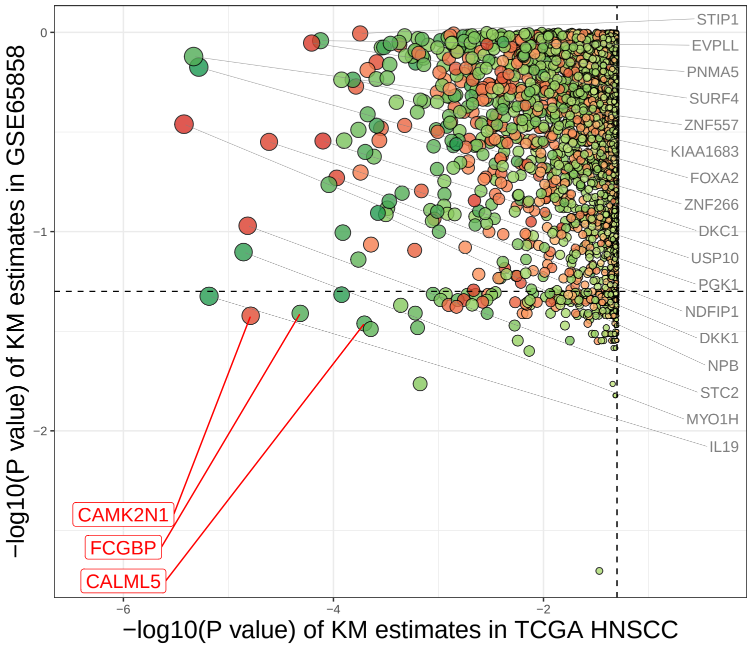

(a) Kaplan–Meier survival estimates from TCGA HNSCC and GSE65858 (Pearson's correlation coefficient[1], r = 0.01).

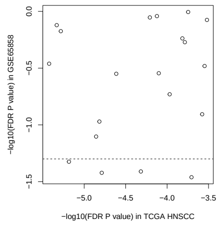

(b) Correlation of Kaplan–Meier survival estimates of those 20 significant genes (Pearson's r = 0.19).

Figure S2. A head-to-head comparison of -log10(FDR-adjusted *P* values) from TCGA HNSCC and GSE65858 datasets. TCGA HNSCC and GSE65858 cohorts were applied for identification and validation of the candidate biomarkers in HNSCC. (a) A total of 5404 genes had FDR-adjusted *P* values of Kaplan–Meier estimates from TCGA HNSCC and GSE65858 (poor Pearson's correlation, r = 0.01). CAMK2N1, CALML5, FCGBP, and 17 genes (marked in black) had FDR-adjusted *P* values < 0.0003 (log10(0.0003) = -3.5) in TCGA HNSCC. Red spots: $HR > 1.0$ in TCGA HNSCC. Green spots: $HR < 1.0$ in TCGA HNSCC. Size of spots: bigger in smaller Kaplan–Meier *P* values in TCGA HNSCC. (b) The 20 genes were extracted and shown. The FDR-adjusted *P* values of those genes have poor correlation between the two cohorts (Pearson's r = 0.19). (X-axis: Kaplan–Meier survival estimates from TCGA HNSCC, with false discovery rate (FDR)-adjusted *P* values (log10 transformed); Y-axis: Those values from GSE65858; TCGA: the Cancer Genome Atlas; HNSCC: head and neck squamous cell carcinoma. Dashed line: 0.05 (or log10-transformed as -1.3)

1. Schober, P.; Schwarte, L.A. Correlation coefficients: Appropriate use and interpretation. *Anesthesia and Analgesia* **2018**, *126*, 1763–1768. doi:10.1213/ANE.0000000000002864.
